# Developing the design of a continuous national health survey for New Zealand

**DOI:** 10.1186/1478-7954-11-25

**Published:** 2013-12-23

**Authors:** Robert Graham Clark, Robert Templeton, Anne McNicholas

**Affiliations:** 1 National Institute for Applied Statistics Research Australia, University of Wollongong, Wollongong, Australia; 2 Health and Disability Intelligence Group, New Zealand Ministry of Health, Wellington, New Zealand

**Keywords:** Health surveys, Indigenous populations, Sample design, Sampling rare populations, Survey planning

## Abstract

**Background:**

A continuously operating survey can yield advantages in survey management, field operations, and the provision of timely information for policymakers and researchers. We describe the key features of the sample design of the New Zealand (NZ) Health Survey, which has been conducted on a continuous basis since mid-2011, and compare to a number of other national population health surveys.

**Methods:**

A number of strategies to improve the NZ Health Survey are described: implementation of a targeted dual-frame sample design for better Māori, Pacific, and Asian statistics; movement from periodic to continuous operation; use of core questions with rotating topic modules to improve flexibility in survey content; and opportunities for ongoing improvements and efficiencies, including linkage to administrative datasets.

**Results and discussion:**

The use of disproportionate area sampling and a dual frame design resulted in reductions of approximately 19%, 26%, and 4% to variances of Māori, Pacific and Asian statistics respectively, but at the cost of a 17% increase to all-ethnicity variances. These were broadly in line with the survey’s priorities. Respondents provided a high degree of cooperation in the first year, with an adult response rate of 79% and consent rates for data linkage above 90%.

**Conclusions:**

A combination of strategies tailored to local conditions gives the best results for national health surveys. In the NZ context, data from the NZ Census of Population and Dwellings and the Electoral Roll can be used to improve the sample design. A continuously operating survey provides both administrative and statistical advantages.

## Background

### Introduction

Health surveys compete for scarce government funds with other priorities, including the direct provision of health services. National health surveys represent a significant expense in absolute terms. Although this expense is dwarfed by the cost of running a national hospital system or widespread health promotion and education activities, surveys still need to demonstrate that they add value to these services commensurate with their cost. Documenting international experience in successful health surveys is essential to make the case for properly resourced national sample surveys and to provide examples of good practice to guide the development of these surveys. This article adds to this literature by describing innovations to the New Zealand (NZ) Health Survey, which moved to continuous operation in 2011. The survey is notable for its quarterly mode of sampling, use of a dual frame design to sample indigenous Māori and other populations, and its potential for linkage to national health administrative datasets. We focus on the survey objectives, general operations, and sample design.

The Health and Disability Intelligence Group of the NZ Ministry of Health manages the survey, including analyses and reporting of results. Following competitive tender processes, the Ministry contracted the field operations for the 2011-2015 period to a survey company, CBG Health Research Ltd, and sample design and technical statistical aspects to the University of Wollongong.

The aim of the survey is to provide timely information on the mental and physical health of New Zealanders, for use in evaluating public policy and to examine the change of possible new public health initiatives. High-level objectives of the survey driving its design include:

i. Monitor the physical and mental health of New Zealanders (both adults and children) and the prevalence of selected long-term health conditions.

ii. Monitor the prevalence of risk and protective factors associated with these long-term health conditions.

iii. Monitor the use of health services and patient experience with these services, including access to services.

iv. Examine differences between population groups, as defined by age, gender, ethnicity, and socioeconomic position.

v. Monitor trends and emerging issues in health-related characteristics including health status, risk and protective factors, and health service utilization.

vi. Measure key health outcomes before and after policy changes and interventions.

To achieve these goals, a continuous survey was developed and has been in the field since July 2011. The survey uses computer-assisted personal interviewing and employs a stratified multistage area sample of approximately 12,500 responding adults and 4,500 children from 12,500 households per year. The annual sample size is approximately the same as the sample size of the previous survey conducted in 2006/2007.

This article describes how a range of tools and strategies were combined to achieve the goals of the continuous survey. The Selective review of national health surveys reviews national health surveys from four countries: the United States, Australia, Canada, and England. Methods used in the NZ Health Survey and the results achieved are described. The main challenge in the sample design was to achieve reasonable precision of estimates for Māori, Pacific, and Asian populations. Sampling of Māori in this survey is also described in more detail in [1], and NZ Ministry of Health reports [2,3] describe the objectives, topics, and sample design of the survey in an expanded form.

### Selective review of national health surveys

#### National health and nutrition examination survey (NHANES)

NHANES has measured the health status and risk factors of the United States for over 50 years [4]. Since 1999, it has been run on a continuous annual basis. The survey consists of an interview to collect household, family, and person-level data and a medical examination including blood tests for all consenting respondents. The sample of 5,000 persons per year is selected by a complex four-stage design, with only 15 primary sampling units (PSUs) (referred to as “stands” and generally corresponding to counties) selected each year. The sample is highly geographically clustered (on average 500 respondents per PSU) to support the medical examination component of the survey. Respondents are asked to attend a mobile examination center, which travels from stand to stand across the year, so that it would be infeasible to select too many stands. Many estimates from NHANES are produced from pooling two years of data, and so are based on 30 PSUs.

Other notable features of NHANES are the use of unequal probability sampling of PSUs in order to give higher probabilities of selection for black American and Mexican-American minority groups. Pregnant women and very young, adolescent, and low-income persons are also oversampled. Multiple respondents are selected from some households, and unequal within-household sampling rates are employed to achieve some of the required oversampling. Surprisingly, the selection of multiple respondents per household apparently improves the response rate, at least for the examination component of the survey.

The use of large and sophisticated mobile examination centers allows high-quality physical measurements to be collected, including vision and dental measurements and blood and urine analysis. A striking example of the value of this approach is the genotyping of a sample of 1991-1994 NHANES respondents, constituting “the first U.S. population-based genetic dataset” [5].

#### Health Survey for England

The Health Survey for England [6] is an annual survey of adults and children, with oversampling of children. It features both an interview with questions on health status and behaviors and a nurse visit with further questions and physical measurements.

The survey has been run annually since 1991, covering adults 16 years and older. Children 2 years and over have been included since 1995 and infants since 2001. The interview is approximately one hour for adults and 20 minutes for children, and includes core questions on health status and related behaviors. Children aged 13-15 are interviewed directly, with permission from a parent or guardian. A parent reported on behalf of children 12 or younger, where possible in the presence of the child. Each annual survey has more detailed questions on a particular topic; in 2010 the focus was on respiratory health.

The survey employs multistage sampling from the so-called small user Postal Address File. This national list of addresses has less than 1% undercoverage and around 9% overcoverage, the latter being due to business and other ineligible addresses. The first stage of selection in 2010 was a sample of 840 PSUs, where PSU populations consisted of at least 500 addresses. Twenty-nine addresses were selected from most PSUs. In most addresses, all adults (up to 10) and two random children were selected. In a minority of households, the adult interviews were omitted as a means of oversampling children within a fixed budget.

The survey consisted of an interview, including measurement of height and weight, and a follow-up visit by a nurse, who asked further questions, took physical measurements including lung function, and collected blood, saliva, and urine samples.

#### National Health Survey 2011-2013 (Australia)

This household interviewer survey of approximately 16,000 households was conducted over 12 months in 2011 and 2012 [7]. Earlier surveys were conducted in 1995, 2001, 2004-2005, and 2007-2008. Information was collected from adults and children on health conditions, well-being, actions, use of private health insurance, and other topics. Height, weight, waist circumference, and blood pressure were also measured by interviewers. Respondents aged 5 years and older could volunteer for the National Health Measures Survey, which involved blood tests (12 years and up) and urine tests (5 years and up) of nutritional status and disease markers, conducted at collection centers.

The National Nutrition and Physical Activity Survey was conducted over approximately the same period. The selected 10,000 households were also asked to volunteer for the National Health Measures Survey. The three combined surveys are referred to as the Australian Health Survey. The Australian Health Survey also includes a further sample of approximately 13,000 Aboriginal and Torres Strait Island people.

The National Health Survey sample design consisted of multistage sampling of households, followed by the random selection of one adult (18 years and up) and one child (if there are any children in the household). The first stage of selection was a sample of collectors districts (an areal unit consisting of approximately 250 dwellings on average), with probability proportional to their size. Selected collectors districts were then divided into blocks, one of which was selected, followed by a systematic sample of households from each block.

#### The Canadian Community Health Survey (CCHS) regional component

Statistics Canada’s CCHS is driven by the need to produce health information for health regions (over 120), provinces and territories (13), and Canada in total. The survey has a two-year cycle and has been running since 2000, with a regional component and a provincial component. See [8], who described a redesign of the survey to take effect from early 2007.

The provincial component is a survey of approximately 30,000 respondents conducted every second year. It produces national and provincial statistics on varying specific topics of current interest.

The regional component is a much larger survey of over 125,000 respondents, with a focus on regional and provincial statistics on health status, service utilization, and determinants of health. It was conducted every second year up to 2006, but from 2007 it has been fielded continuously, with each six-month period comprising a nationally representative sample. These periods can then be aggregated as appropriate for differing regions and variables. The continuous survey model was adopted to allow timely response to emerging data requirements and to stabilize Statistics Canada’s interviewing workloads.

One unique feature of the regional component is its flexible content. The 45-minute interview is divided into: (a) 30 minutes of core questions asked of all respondents; (b) 10 minutes of content specific to each region chosen from a portfolio of content modules; and (c) one of three five-minute question modules, each applied to approximately one-third of respondents (randomly selected).

A dual frame sample design is used. Approximately one-half of the sample is selected from a list of telephone numbers by stratified simple random sampling with health regions as strata. Random digit dialing was initially used but replaced by sampling from a frame of telephone numbers (except in three remote provinces) due to low hit rates (although higher coverage). The other half of the sample of dwellings is a multistage area-based sample.

The final stage of selection was of one person (aged 12 years and older), with unequal probabilities based on age. This was done partly to make up for the undersampling of 12- to 19-year-olds, which was found to result from selecting one person per household.

## Methods

### Overview

The sample design is complex, involving use of the NZ Electoral Roll to oversample Māori and data from the 2006 NZ Census of Population and Dwellings (NZ Census) to target a general area-based sample. The first step was to set the annual sample size. The responding sample size is approximately 12,500 adults per year. This figure was chosen based partly on budget constraints and partly on target standard errors for Māori and national prevalences (see Tables one and two of [3]). The New Zealand Health and Disability Multi-Region Ethics Committee granted approval for the survey (MEC/10/10/103) in 2011.

The sample was made up of two components. Area-based sample summarizes the general area-based sample. This sample was supplemented with a sample of those households where at least one adult indicated Māori descent on the NZ Electoral Roll. The covered population of the Roll component was a subset of that of the area-based component. The two parts of the sample were constrained to consist of non-overlapping sets of meshblocks.

### Area-based sample

#### Probability proportional to size sampling of the primary sampling units (PSUs)

The area-based design was a multistage sample, stratified by District Health Board (DHB). At the time of design, there were 21 District Health Boards in New Zealand, with 2006 NZ Census of Population and Dwellings (NZ Census) populations ranging from approximately 30,000 (West Coast) to 460,000 (Waitemata). DHBs are responsible for provision of health services in their district.

In a stratified design, the allocation of the total sample to strata needs to be determined. If only national statistics are important, a roughly proportional allocation, with stratum sample sizes proportional to population sizes, is usually close to optimal. However, it is also important to be able to calculate statistics for each DHB with reasonable precision, even for the smaller districts. As a compromise between both concerns, stratum sample sizes were calculated to be proportional to the square root of the 2006 census population for the stratum in a power allocation [9].

A multistage sample design is used to reduce travel and listing costs. The PSU was the meshblock, an area unit consisting of on average about 40 households but with a wide variation in size (coefficient of variation of about 70%). Within each stratum, a sample of meshblocks was selected, followed by a sample of dwellings in the selected meshblock, followed by a randomly selected adult and child (if any) in selected dwellings.

In a standard self-weighting multistage design, PSUs are selected from each stratum with probability proportional to their population size (PPS) (according to the census number of dwellings), and the same number of dwellings is then selected from every selected PSU. This results in all households in a stratum having equal chance of selection in the survey, which is usually statistically efficient in the sense of achieving low standard errors, and which may also simplify the calculation of weights used in producing estimates from the survey. This approach was modified to give higher probabilities for households in areas where Māori, Pacific, or Asian people are more prevalent. Let $N_{i}^{*}$ be the population in meshblock (MB) *i* according to the 2006 NZ Census. The probability assigned to MB *i* is

(1) $$\pi_{i}=m_{h}N_{i}^{*}f_{i}/\left(\sum_{i\in h}N_{i}^{*}f_{i}\right)$$

where *m*_*h*_ is the required sample size of meshblocks in DHB *h*, and *f*_*i*_ is a “targeting factor” by which areas with more Pacific or Asian people are oversampled.

The targeting factor is given by a weighted average of the square roots of the Pacific and Asian densities at meshblock and Area Unit (AU) levels (according to the 2006 Census) and a constant. AUs are a geographic unit consisting of groups of MBs; there are approximately 1900 AUs containing on average about 800 occupied dwellings. The targeting factor was calculated as

(2) $$\begin{array}{c} f_{i}=0.31\sqrt{\text{Pacific}\;\text{MB}\;\text{density}}+0.37\sqrt{\text{Pacific}\;\text{AU}\;\text{density}}\\ \;+0.09\sqrt{\text{Asian}\;\text{MB}\;\text{density}}+0.20\sqrt{\text{Asian}\;\text{AU}\;\text{density}}+0.03 \end{array}$$

This definition of the targeting factor was designed to target the sample toward areas with higher proportions belonging to the subpopulations of interest, while reflecting the fact that making selection probabilities too unequal carries a penalty to standard errors. The use of square roots of densities was motivated by the optimal designs of [10] and [11]. The factor was based on both meshblock and AU densities, because the former gives a more locally targeted sample but is more sensitive to the outdatedness of the census data, while the latter is more stable over time. The imperfections in the census data are particularly important because the census was about five years out of date in mid-2011. The coefficients in *f*_*i*_ were obtained by numerical optimization to give the lowest possible estimated standard errors, where this estimation used 2006/2007 NZ Health Survey data to evaluate designs based on 2001 Census data with given coefficient values. For a detailed explanation of this approach, see Appendix 1 of [3], and [1]. See also [12] for a discussion of a generalized method of sample allocation allowing for imperfections in the design data.

#### Selecting households from the selected PSUs

An equal probability sample of households is selected from each selected meshblock, with sampling fraction of $c/N_{i}^{*}$, where c is the target within-MB sample size. If the MB population is still the same as in the census, then c households are selected. In practice, meshblock populations have usually changed from $N_{i}^{*}$. Let *H*_*i*_ be the number of dwellings in meshblock i at the time of enumeration, then $H_{i}c/N_{i}^{*}$ dwellings are selected. Unpublished research suggests that changes in population size since 2006 to 2012 may have a similar effect on sampling efficiency as changes in ethnicity densities (A. Gray, personal communication October 31, 2013), and this will be investigated when the survey is redesigned after its fourth year.

The target within-PSU sample size, c, is a trade-off of cost and sampling error (e.g., see Chapter 6 of [13]). If c is large, then the sample is highly clustered, so that relatively few MBs need to be selected to achieve a given sample size of households. This reduces interviewer travel costs but increases sampling error. The converse applies when c is small.

The best value of c depends on the variable to be estimated, in particular its “intra-class correlation” (a measure of how geographically clustered the variable is). The higher the intra-class correlation, the smaller the target cluster size should be, and therefore a lower value of c is needed.

The value of c has been set at 20. This value is larger than is common for many surveys, but is thought to be appropriate for the NZ Health Survey for the following reasons.

• Intra-class correlations for many rare health condition variables are small, so that a larger cluster size is appropriate.

• Cluster sizes for subpopulations such as Māori, Pacific, or Asian people are generally significantly smaller than 20.

• A cluster size of 20 would mean that a significant proportion (roughly one-third, on average) of the meshblock needs to be used. A high sampling fraction within meshblocks implies fewer meshblocks in sample. This is desirable in order to control for the overlap of meshblocks with other surveys and to reduce listing costs.

The net result of the sampling of MBs and this sampling method within MBs is that household probabilities of selection will be proportional to the targeting factor, *f*_*i*_, within each stratum. The sample design could be described as “lightly targeted.” Households in areas with a higher Pacific and Asian population are given a higher chance of selection, but not dramatically so, due to the square root sign in formula (2) and also due to the use of the broader area units’ densities in (2). It is well known that overtargeted designs can lead to higher sample sizes but worse precision for subpopulations (see [10], page 9 of [14], and [15]).

#### Selection within households

The final stage of selection is to list all adults and children in each household, and to select a random adult (15 years and over) and child (0-14 years, if any). The survey interview is on average approximately one hour for adults and 30 minutes for adults reporting on behalf of a child, so selecting more than one adult and one child would be overly burdensome on respondents.

### List-based sample from electoral roll

A stratified two-stage sample of addresses is selected quarterly from the Electoral Roll in order to increase the number of Māori in sample and to reduce the standard errors of statistics on the Māori population. The addresses selected are those where a person has self-identified as having Māori ancestry on the Electoral Roll, regardless of whether they are enrolled in general or Māori seats. The first stage of selection is a stratified sample of meshblocks with probability proportional to the number of these addresses on the Electoral Roll in the meshblock. Strata are defined by DHB. The second stage of selection is a random sample of 10 addresses from each selected meshblock (or all addresses, if less than 10). The sample of meshblocks will be non-overlapping with the area-based sample.

As with the area based sample, one adult (15 years and over) and one child (0-14 years, if any) are selected at random from each selected address. The selection within households is made without reference to the Electoral Roll and Māori individuals are not preferentially selected, in order to ensure that correct probabilities of selection can be calculated for all respondents. Addresses with multiple people with identified Māori ancestry were not treated any differently.

Approximately 15% of selected households were selected from the Electoral Roll, with the remainder obtained from the area-based sample. This and other features of the sample design will be retained for the first four years of the continuous survey and then reviewed.

### Other sample design features

#### Weighting

The final sample was given by pooling the area and roll samples. Probabilities of selection (defined as the probability of being in the pooled sample) were calculated for all respondents and were the basis of the weights used to calculate all survey estimates. Even though both parts of the sample, particularly the Roll component, were designed to give increased chance of selection to Maori, Pacific, and Asian respondents, the use of weights ensures that estimates are unbiased.

#### Proxy screening

In the 2006-2007 NZ Health Survey design, a proxy screening process was used where one adult reported on the ethnicity of all household members. This information was used in selecting an adult and child from each household in order to give higher probabilities of selection to Māori, Pacific, and Asian people. Proxy screening was dropped for the continuous survey because around 20% of Māori were not identified using this approach in 2006/2007. Moreover, asking a householder to report the ethnicity of all residents before a person was selected for interview likely created resistance to survey participation. For more information on this issue, see [1].

#### Institutions

Residents of rest homes, excluding psychiatric and dementia care units, are also in scope of the survey. Rest homes in selected PSUs in the area-based sample are divided into accommodation units which typically consist either of individuals or couples living together. Accommodation units are then treated as households for the purpose of sample selection. Students living away from home in university hostels and boarding schools are selected via their family’s house, if they still consider this to be their home. Arrangements are made to survey them either when they are next at home or at their current residence.

#### Allocation to quarter

The samples of meshblocks are selected on an annual basis and randomly assigned to a quarter for enumeration to avoid any bias due to seasonality of health variables and to enable valid estimates to be calculated from each quarter.

### Survey content

The interview component of New Zealand’s health survey collects information on respondents’ perception of the accessibility and quality of services, risk and protective factors, self-assessment of their health, and other information not available from administrative data. The survey interview also enables controlled and stable definitions across time, whereas administrative datasets can be subject to changes to meet different service delivery and policy priorities.

The questionnaire includes a set of “core” questions drawn from each of nine information domains. These core questions will be the same each year and make up about half of the survey questions. The survey also includes questions that examine a topic in more depth. These “module” questions will change each year and will make up the other half of the survey questions. The information domains covered by the survey, under which both the core and module questions fall, include the following:

• long-term health conditions

• risk and protective factors (including physical activity, tobacco use, alcohol consumption, drug use, problem gambling, and sexual and reproductive health)

• nutrition

• mental health

• oral health

• health service utilization

• patient experience

• social determinants of health.

The module topics for the first year (2011-2012) were health service utilization and patient experience in adults and children and problem gambling and racial discrimination in adults. The second year’s module topics included tobacco, alcohol, and drug use, and child development and well-being, and in the third year, long-term conditions. A sexual and reproductive health module is planned for 2014-2015.

Taking tobacco consumption to illustrate the concept of core and module questions, the survey includes nine core questions regarding smoking status such as having ever smoked, frequency of current smoking, the number of cigarettes smoked per day, and for ex-smokers the time since cessation. When the tobacco use module was fielded during the survey’s second year of operation, an additional approximately 35 questions were added. These module questions included topics such as age of smoking initiation, awareness and use of different cessation programs and products, second-hand smoke exposure, and smoking during pregnancy.

A key strategy of the survey is the use of objective measurements where practicable. For example, height and weight are core measurements undertaken by the interviewer, the former using a laser height device found to be more reliable than a stadiometer method. Blood pressure measurement in adults was introduced as a core measure in the survey’s second year. Blood and urine samples will be collected periodically starting in mid-2014. Participants will go to their local medical laboratory to have specimens taken. Response rates for the main survey are not expected to be affected because the tests will be a separate voluntary step after the interview has been completed.

Combing data from health surveys with administrative heath data from a range of sources, e.g., hospitalization data and cancer registries, can increase substantially the range of heath-related topics that can be investigated and allows more complex health issues to be examined. The NZ Health Survey explicitly seeks consent from participants to link their survey data to routinely collected administrative health datasets, and they sign a separate consent form to allow such data linkage at the end of the interview. Identifying details such as name and address are used to match participants to their National Health Index Number, a unique identifier used within the NZ health system. To protect the participant’s confidentiality, this linkage process occurs independently of survey responses; that is, the person performing the data linkage does not have access to the associated survey information, and only an encrypted National Health Index Number is added to the survey dataset.

## Results and discussion

### Advantages of the continuous nature of the survey

Set-up and project management costs were previously duplicated across the three-yearly health surveys and the separate topic-specific surveys fielded in the years between health surveys; for example, adult and child nutrition surveys and oral health surveys. Undertaking a single competitive procurement process to select a survey provider for one combined survey (for an initial period of approximately five years) significantly reduces transaction costs and overheads for both the Ministry and survey providers. This approach creates the potential to increase the stability and quality of the survey field workforce, resulting in better quality data.

In the past, the sample size was approximately 12,000 adults and 4,000 children for the three-yearly health survey with collection generally spread over a calendar year. The new continuous survey involves approximately 12,500 adults and 4,500 children annually, which suggests a higher cost over time. However, this cost is contained by the inclusion of the separate topic-specific surveys (previously fielded in the years between health surveys) as rotating modules in the new health survey.

With a continuous survey it will also be possible to pool survey datasets across multiple quarters or years. Pooling datasets will improve both the statistical precision of estimates for Māori and ethnic minorities (including Pacific and Asian ethnic groups) and the range and statistical quality of analyses that can be undertaken at regional or district level.

Rather than collecting data in great detail but relatively infrequently (as previous surveys did), a continuous survey will allow the more frequent collection of less detailed data on a topic. Information “packages” could be produced each year based on annual core datasets, with the comprehensive, detailed information packages from each topic module produced separately.

Each quarter’s sample is a representative probability sample so that valid quarterly estimates can be produced. Quarterly samples will be relatively small, so that standard errors will be high, but quarterly estimates will still be useful, because they will enable pooling over an appropriate number of quarters for producing statistics on a given topic. In general, one or more whole years of data will be used to average out seasonality effects. The availability of quarterly estimates will also make it possible to analyze quarterly time series, for example using seasonal adjustment or trend estimation using an exponentially weighted or other moving average (e.g., page 106 of [16]), both of which are widely used in subannual surveys conducted by national statistics offices [17]. Pooling data over, say, two years represents a very simple way of estimating the trend of a data series. A more sophisticated version would consist of an exponentially weighted average of eight or more quarters with more weight given to recent quarters. This and other time series analyses, including the choice of smoothing parameters to trade off timeliness and stability, will be investigated once three years of data are available.

### Respondent cooperation

The continuous survey has now been in the field for more than two years, with high response rates maintained, and three topic modules successfully rotated into the survey. These module rotations included the successful implementation of a Computer Assisted Self Interview (CASI) to allow participants to self-complete the sensitive questions within the tobacco, alcohol, and drug use module. During the 2011/2012 survey period, the response rate for adults was 79%. The response rate was calculated using definition RR3 on page 45 of [18], which includes an estimated eligibility rate for non-contacts whose eligibility is uncertain, as described in Section 5 of [19]. This is a particularly good result from a non-compulsory survey, which we attribute to a combination of the face-to-face interview mode of collection, a well-qualified and experienced interviewer panel, the use of up to 10 call-backs in the case of respondents not at home, the perceived usefulness of the survey, and perhaps the congeniality of discussing one’s own health. Over 90% of survey respondents further consented to data linkage using their National Health Index Number, which is also an outstanding accomplishment. The use of this identifier has meant that linkage is virtually always successfully achieved for consenting respondents. Details of the use of the linked administrative variables in statistical outputs are still under development.

### Efficiency of the sample design

The design effect (deff) is a measure of the efficiency of a sample design (e.g. [20]). It is the ratio of the variance of a statistic of interest to the variance that would be achieved by a simple sample design (typically simple random sampling) with the same sample size. Table 1 shows prevalences and design effects for 11 key indicators from the survey for all adults (15 years and over) and for Māori and Pacific adults. Design effects were estimated by using the jack-knife replicate weights produced by the NZ Ministry of Health to capture the complex sample design. The sample design is multistage with households clustered in meshblocks, and the intra-class correlations within meshblocks are also shown.

**Table 1:** Results for key indicators by ethnicity from year 1 of the survey

**Indicator**	**All ethnicities**				**Māori**				**Pacific**			
	**Prevalence (%) (a)**	**SE (%) (b)**	**Intra-class correlation (c)**	**Design effect (d)**	**Prevalence (%) (a)**	**SE (%) (b)**	**Intra-class correlation (c)**	**Design effect (d)**	**Prevalence (%) (a)**	**SE (%) (b)**	**Intra-class correlation (c)**	**Design effect (d)**
Excellent, very good, or good self-rated health	89.3	0.37	0.02	1.80	83.4	0.93	0.02	0.99	86.6	1.68	0.02	1.77
Current smoking	18.4	0.49	0.04	1.97	40.6	1.42	0.04	1.32	26.2	1.83	0.04	1.27
Meets vegetable (3+ servings) and fruit (2+ servings) daily intake guidelines	44.2	0.87	0.06	3.84	37.1	1.25	0.06	1.06	29.9	2.49	0.06	2.16
Physically active	54.4	1.27	0.21	8.15	58.1	1.71	0.21	1.88	47.4	2.60	0.21	1.97
Obese	28.4	0.62	0.03	2.03	44.2	1.34	0.03	0.98	61.7	2.78	0.03	2.03
High blood pressure (medicated)	15.8	0.40	0.01	1.48	13.2	0.83	0.01	0.96	10.9	1.10	0.01	0.91
Diagnosed depression, bipolar disorder, and/or anxiety disorder	16.2	0.46	0.02	1.98	16.1	1.02	0.02	1.23	6.8	1.05	0.02	1.27
Diagnosed diabetes	5.5	0.24	0.02	1.35	7.4	0.66	0.02	1.00	10.3	1.14	0.02	1.03
Visited a GP in the past 12 months	78.5	0.49	0.02	1.79	75.2	1.35	0.02	1.56	75.7	2.04	0.02	1.65
Experienced unmet need for primary health care in the past 12 months	26.6	0.61	0.07	2.42	39.1	1.44	0.07	1.38	30.3	2.33	0.07	1.87
Visited a dental health care worker in past 12 months (dentate adults)	48.6	0.67	0.04	2.00	37.6	1.59	0.04	1.49	32.8	2.07	0.04	1.24

a: calculated using survey weights.

b: calculated using jack-knife replicate weights reflecting complex sample design and weighting, as used in production of standard errors in survey publications.

c: estimated by fitting a linear mixed model using the lme4 package in the R statistical environment.

d: calculated by dividing b by the estimated variance under simple random sampling from the whole adult population.

All but three of the design effects are larger than 1. This is due to various sampling techniques that improve subpopulation statistics, reduce survey cost, and improve respondent cooperation, but which incur a penalty to standard errors. The average design effects over the 11 indicators were 2.00, 1.49, and 1.24, for all, Māori, and Pacific adults, respectively. The design effects for all-ethnicity estimates are higher because the sample of all adults is more clustered (i.e., more respondents per meshblock), and also because the oversampling of Māori, Pacific, and Asian adults leads to greater variation in weights when all adults are pooled than when just one ethnicity is considered. The largest design effects occurred for the Physically Active indicator, due to its high intraclass correlation (0.21 for all adults). These values for the design effects are not unusual in an area-based household survey, see for example [21].

The design effects vary substantially across indicator and subpopulation. Table 2 shows approximate design effects due to different components of the design. If the factors shown were the only ones in play, the product of these components would equal the design effects shown in Table 1. This was not the case, as there were many other factors, including the variable-specific effects of clustering and the use of calibrated weighting. An Appendix describes the method of calculating the values in Table 2.

**Table 2:** Components of design effect associated with sample design

**Component of design effect**	**All ethnicities**	**Māori**	**Pacific**	**Asian**
Varying weights due to disproportionate allocation to strata (a)	1.12	1.13	1.06	1.06
Over- or undersampling due to disproportionate allocation to strata (b)	1.00	0.90	1.13	1.19
Varying weights due to 1/household sampling (c)	1.29	1.33	1.37	1.30
Over- or undersampling due to 1/household sampling (d)	1.00	1.00	1.25	1.15
Varying weights due to unequal probabilities of selection of households within strata (e)	1.17	1.14	1.08	1.06
Over- or undersampling due to unequal probabilities of selection of households within strata (f)	1.00	0.71	0.68	0.91
Unequal probability of selection of households within strata (product of e and f)	1.17	0.81	0.74	0.96

Rows (a) and (b) show the effect of the disproportionate allocation to strata (District Health Boards), where the strata sample sizes from the area frame are proportional to the square root of the population sizes. This allocation is a compromise between national efficiency and precision for smaller strata. It results in variation in weights, undersampling of the Pacific and Asian populations (because these populations are somewhat concentrated in larger strata), and oversampling of the Māori population (because this population is somewhat concentrated in smaller strata).

Row (c) of Table 2 shows that much of the design effect is due to the variation in estimation weights caused by the sampling of just one adult per household. Row (d) shows another effect of one-per-household sampling, namely that it results in undersampling of ethnic populations whose average house size is larger. This has little effect on Māori statistics, but increases variances of Pacific and Asian statistics by 25% and 15%, respectively.

Rows (e) and (f) show the effect of using disproportionate area sampling within strata and the dual frame design. Row (e) shows that there is an increase to the design effect due to the greater variation in selection probabilities arising from the use of these tools. However, for the ethnic subpopulations, this is more than counter-balanced by the increased sample take of the subpopulation, as shown in (f). Taking the product of (e) and (f) tells us that disproportionate sampling within strata increases the variance of all adult statistics by approximately 17% and decreases the variances of Māori and Pacific statistics by approximately 19% and 26%, respectively. However, the improvement in Asian statistics was only 4%, in spite of the targeting toward areas with more Asian residents in formula (2) of the Methods section. Further investigation showed that across all meshblocks in NZ, the 1996 proportion of people who were Asian in the meshblock had correlations of 0.14 and -0.15 with the proportions who were Pacific and Māori. Oversampling of areas with more Māori residents appears to have the unintended consequence of almost removing the oversampling of the Asian subpopulation.

## Conclusions

Compared to the other national health surveys discussed, the NZ Health Survey was the only one set up to allow quarterly statistics. It has a relatively large sample size, includes direct measurements (although not currently to the extent of NHANES or the Australian Health Survey), and allows data linkage with health administrative datasets. It maintains a strong focus on Māori statistics through the use of an innovative dual frame design. The design combines a multistage area sample with unequal probability sampling of areas and a list-based sample from addresses on the Electoral Roll where a resident has indicated Māori descent. The survey has had a successful first two years of operation, maintaining high response rates and introducing changing topic modules.

What lessons does this survey have for other existing or planned national health surveys? Firstly, the considerable effort in setting up a survey that runs continuously is balanced by statistical, personnel, and operational advantages. Results can be reported more frequently. The inclusion of rotating topic modules provides flexibility and contains costs, and the survey’s continuous operation provides unique opportunities to make ongoing improvements. Secondly, ethnic and indigenous subpopulations can be sampled effectively using a dual frame approach and unequal probability sampling by area in the area-based component. The details will vary from country to country depending on the availability and quality of population lists. Careful planning is needed so that targeting by region and sampling from a list are combined in a manner reflecting the differing imperfections of both sampling methods.

## Appendix

### Calculation of design effect components

Applying Kish’s well-known rule [22] for the design effect due to unequal probabilities of selection, assuming no effect due to clustering, and rewriting in terms of the coefficient of the variation of the weights gives:

(A1) $$\mathrm{var}\left(\hat{\bar{Y}}\right)\approx \frac{1+C_{w}^{2}}{n}S_{Y}^{2}$$

where $\hat{\bar{Y}}$ is an estimated mean or prevalence, $\mathrm{var}\left(\hat{\bar{Y}}\right)$ is its variance under a complex sample design, *C*_*w*_ is the sample coefficient of variation of the estimation weights which are assumed to be the inverses of the probabilities of selection, $S_{Y}^{2}$ is the population variance of *Y*, and *n* is the sample size. For estimates of the mean of a subpopulation (e.g. Māori), we have

(A2) $$\mathrm{var}\left({\hat{\bar{Y}}}_{\mathrm{sub}}\right)\approx \frac{1+C_{\mathrm{sub}}^{2}}{n_{\mathrm{sub}}}S_{\mathrm{sub}}^{2}$$

where *n*_*sub*_ is the sample size from the subpopulation, $C_{\mathrm{sub}}^{2}$ is the coefficient of the variation of the weights for just subpopulation respondents, and $S_{\mathrm{sub}}^{2}$ is the population variance of *Y* for just the subpopulation.

Let design (a) be simple random sampling of all people; (b) be simple random sampling of all people within strata where stratum sample sizes are proportional to the square roots of the population sizes; (c) be simple random sampling of households within strata with one person selected from each household, and stratum sample sizes are proportional to the square roots of the population sizes; and (d) be the actual design with unequal probability sampling of households within strata, stratum sample sizes proportional to the square roots of the population sizes, and one person selected per household. We define all four designs to have the same sample size of people (including subpopulation members and non-members). The design effect is

(A3) $$\mathrm{deff}=\frac{\mathrm{var}\left({\hat{\bar{Y}}}_{\mathrm{sub}}:\mathrm{design}\;c\right)}{\mathrm{var}\left({\hat{\bar{Y}}}_{\mathrm{sub}}:\mathrm{design}\;a\right)}\approx \frac{1+C_{\mathrm{sub}\left(d\right)}^{2}}{n_{\mathrm{sub}\left(d\right)}}/\frac{1+C_{\mathrm{sub}\left(a\right)}^{2}}{n_{\mathrm{sub}\left(a\right)}}$$

where the subscript (x) refers to the values that would be expected from design x, where x may be a, b, c, or d.

We can expand (A2, A3) as

(A4) $$\begin{array}{l} \mathrm{deff}\approx \left(\frac{1+C_{\mathrm{sub}\left(b\right)}^{2}}{1+C_{\mathrm{sub}\left(a\right)}^{2}}\right)\;\left(\frac{1+C_{\mathrm{sub}\left(c\right)}^{2}}{1+C_{\mathrm{sub}\left(b\right)}^{2}}\right)\\ \;\times \left(\frac{1+C_{\mathrm{sub}\left(d\right)}^{2}}{1+C_{\mathrm{sub}\left(c\right)}^{2}}\right)\;\left(\frac{n_{\mathrm{sub}\left(a\right)}}{n_{\mathrm{sub}\left(b\right)}}\right)\;\left(\frac{n_{\mathrm{sub}\left(b\right)}}{n_{\mathrm{sub}\left(c\right)}}\right)\\ \;\times \left(\frac{n_{\mathrm{sub}\left(c\right)}}{n_{\mathrm{sub}\left(d\right)}}\right) \end{array}$$

The four factors in (A4) give the decomposition in Table 2. All that remains is to assign values to the quantities in (A4). We have data from a sample selected using design (d), which we will denote *s*_*d*_. Formula (2) in Section 6.3 of [1], and the discussion following it, show that we can estimate the sample size that will be achieved from any design x using

(A5) $${\hat{n}}_{\mathrm{sub}\left(x\right)}=\sum_{s_{d}}w_{\mathrm{di}}\pi_{\mathrm{xi}},$$

and $1+C_{\mathrm{sub}\left(x\right)}^{2}$ can be estimated using

(A6) $$1+{\hat{C}}_{\mathrm{sub}\left(x\right)}^{2}=\frac{\sum_{s_{d\left(\mathrm{sub}\right)}}w_{\mathrm{di}}\pi_{\mathrm{xi}}\sum_{s_{d\left(\mathrm{sub}\right)}}w_{\mathrm{di}}\pi_{\mathrm{xi}}^{-1}}{{\left(\sum_{s_{d\left(\mathrm{sub}\right)}}w_{\mathrm{di}}\right)}^{2}}$$

where *w*_*di*_ is the survey weight, and *π*_*xi*_ is the probability of selection for sampled unit i under design x. The values of *π*_*xi*_ can readily be obtained from variables on the survey file, as the household size, stratum population sizes, and the probability of selection under design d are all available.

## Competing interests

The first two authors are employed by the NZ Ministry of Health, and the third author is employed by the University of Wollongong, which has been contracted to provide statistical support to the NZ Ministry of Health.

## Authors’ contributions

RC led the sample design and weighting of the survey and was the lead writer of this paper. RT participated in the design, statistical analysis, strategic planning and management of the survey, and participated in the preparation of this manuscript. AM led the development of the survey instruments and management of the survey and participated in the preparation of this manuscript. All authors read and approved the final manuscript.
